# Therapeutic potential of regulatory T cells for stem cell regulation: Insights from Treg-mediated enhancement of limbal stem cell functions

**DOI:** 10.1016/j.isci.2025.112515

**Published:** 2025-04-22

**Authors:** Fei Fang, Tingxi A, Junzhao Chen, Shiding Li, Tianyi Zhou, Liangbo Chen, Yao Fu, Chunyi Shao

**Affiliations:** 1 Department of Ophthalmology, Shanghai Ninth People’s Hospital, Shanghai JiaoTong University School of Medicine, Shanghai, P.R. China; 2 Shanghai Key Laboratory of Orbital Diseases and Ocular Oncology, Shanghai, P.R. China

**Keywords:** Therapeutics, Immunology, Stem cells research

## Abstract

Regulatory T cells (Tregs) play a key role in immunomodulation and tissue regeneration. Limbal stem cells (LSCs) maintain corneal epithelial homeostasis, and LSC deficiency (LSCD) leads to visual impairment. Current LSCD treatments face donor shortages and graft rejection risks. The present study explored Tregs’ therapeutic potential for LSCD in a mouse model of graded LSCD and further explored the direct effect of Tregs on LSCs function by *in vitro* coculturing human-derived cells. Subconjunctival Tregs injection effectively treated mild and moderate LSCD in mouse models. Coculturing human LSCs with human Tregs promoted LSCs migration, proliferation, and stemness maintenance. Furthermore, amphiregulin (AREG), secreted by Tregs, was crucial to their therapeutic effects, as AREG^−/−^ Tregs resulted in diminished efficacy on LSCD mice compared to wild-type Tregs. These findings highlight Tregs as a promising treatment for LSCD, enhancing LSC function partially via AREG production.

## Highlights

• Tregs injection effectively treats mild and moderate LSCD in mice
• Tregs enhance LSC migration, proliferation, and stemness *in vitro*
• Amphiregulin is crucial for Tregs’ therapeutic effects on LSCD

## Graphical abstract

**Figure:**
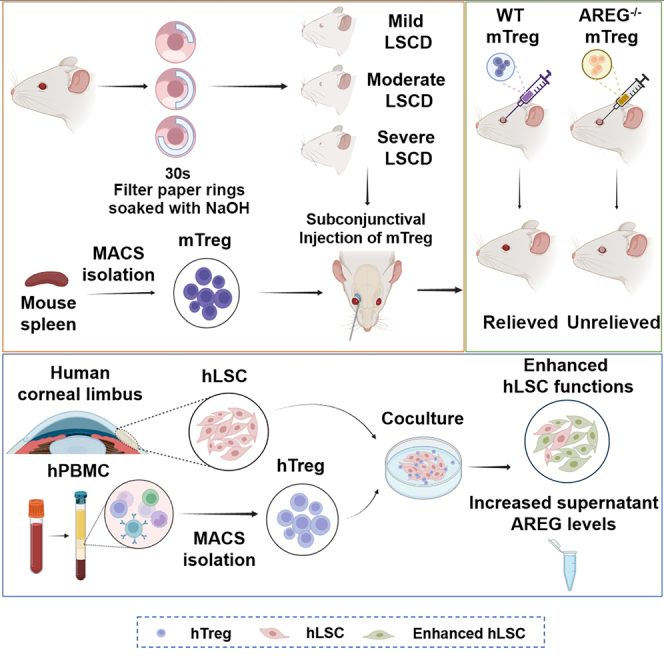

## Introduction

The ocular surface tissue comprises the cornea, the conjunctiva, the eyelids, the meibomian glands, and the lacrimal gland. Exposed to the environment, the ocular surface acts as the external barrier of the eyeball.^1^ Additionally, as a mucosal tissue equipped with immune cells and related factors, the ocular surface can fight against pathogens through innate and adaptive immune responses and prevent unnecessary or excessive inflammatory reactions.^2^

The corneal epithelium, the most superficial layer of the cornea, possesses high regenerative capacity. Limbal stem cells (LSCs) are indispensable for corneal epithelium regeneration and corneal wound healing.^3^ Putative markers of LSCs include ΔNp63α^+^, ABCG2^+^, ABCB5^+^, K12^−^, K14^+^, K15^+^, K19^+^ N-cadherin^+^,^4^ BCAM^+,^^5^ GPHA2^+^,CD63^+^, IFITM3^+^,^6^ and S100A2^+^.^7^ LSCs harbor in the LSC niche and typically remain quiescent during the steady state of the corneal epithelium.^8^ However, they exhibit symmetric or asymmetric division capacity in response to diverse environmental cues.^9^ The centripetal migration and differentiation of LSCs are fundamental processes ensuring the continuous replenishment of corneal epithelial cells, thereby maintaining the dynamic equilibrium within the corneal epithelium.^10^^,^^11^ The niche is a protective microenvironment provided by vault-like structures, including limbal crypts, limbal lacunae, and palisades of Vogt in human tissue. The niche also furnishes the necessary factors to maintain stemness properties of LSCs and initiate differentiation pathways.^12^ Loss and/or damage of LSCs, clinically defined as LSC deficiency (LSCD), leads to disorder of the limbal barrier, defective epithelial regeneration, flawed wound healing, corneal neovascularization, and scarring.^13^ Patients frequently encounter persistent symptoms, such as chronic conjunctival redness, diminished vision, a sensation of foreign bodies, excessive tearing, blepharospasm, and recurrent episodes of pain associated with recurrent epithelial breakdown. The pain, along with photophobia and discomfort, can be enervating for patients.^13^

Regulatory T cells (Tregs) are a group of T lymphocytes expressing CD4, CD25, and forkhead box P3 (FOXP3) that comprise 5–7% of total CD4^+^ T cells. They develop in the thymus (tTreg) or can be induced in the periphery (pTreg).^14^ They can also be *in vitro* induced in cell culture (iTreg) upon exposure to transforming growth factor β (TGF-β).^15^ Their ability to maintain immune tolerance and homeostasis was first demonstrated by Sakaguchi et al. in 1995.^16^ After decades of research, cellular therapies using Tregs are currently undergoing clinical trials for the treatment of graft-versus-host disease (GVHD),^17^ solid organ transplant rejection,^18^^,^^19^^,^^20^ type 1 diabetes,^21^ and autoimmune diseases including systemic lupus erythematosus (SLE),^22^ and inflammatory bowel disease (IBD).^23^ Similarly, Tregs are part of the immune compartment of the ocular surface, and they actively suppress abnormal or excessive immune responses against self, microbial, and environmental antigens to regulate the ocular surface microenvironment.^24^ Beyond their well-established function in immune regulation, more recently, Tregs have been demonstrated to maintain tissue homeostasis and enhance the repair of impaired tissues, such as the skin,^25^ cardiac muscle,^26^ lungs,^27^ and the central nervous system.^28^ Mechanistically, it has been shown that Tregs promote tissue repair under inflammatory conditions by secreting tissue-remodeling proteins, such as amphiregulin (AREG).^29^

Our group has extensively studied the immunoregulatory function of Treg in cornea. Shao et al. have shown that subconjunctivally injected Tregs could migrate to the cornea, regulate the host immune response against corneal allograft, and promote allograft survival in a murine model of corneal transplantation.^30^ Yan et al. have found that the subconjunctival injection of Tregs also effectively promotes corneal wound healing by suppressing excessive inflammation and improving epithelial renewal in a mouse model of corneal alkali burn, evidenced by those treated with Tregs showing significantly lower corneal opacity score, less edema, and accelerated re-epithelialization compared to the control group.^31^

Recently, Altshuler et al. found that Tregs were present at the outer margin of the corneal limbus. After the subconjunctival injection of an anti-CD25 antibody to deplete limbal Tregs, markers of quiescent LSCs significantly declined. It was thus proposed that Tregs, as a constitutional component of the limbal niche, play a critical role in maintaining LSC quiescence, controlling epithelial thickness, and promoting epithelial wound healing.^6^ However, the direct interaction between Tregs and LSC has yet to be explored.

In this study, using our established murine model of injury-induced LSCD, we evaluated the therapeutic efficacy of subconjunctival injection of Tregs in treating LSCD with varying degrees of severity. Furthermore, we co-cultured human-derived Tregs with LSC for the first time to explore the direct effects of Tregs on LSC function using combined cellular assays and RNA sequencing (RNA-seq). Our data demonstrate that Treg-based cellular therapy effectively improves LSCD-associated ocular damage by promoting LSC migration, stemness, and proliferation.

## Results

### Subconjunctival injection of Tregs is effective in treating mild and moderate, but not severe LSCD

**Figure 1:**
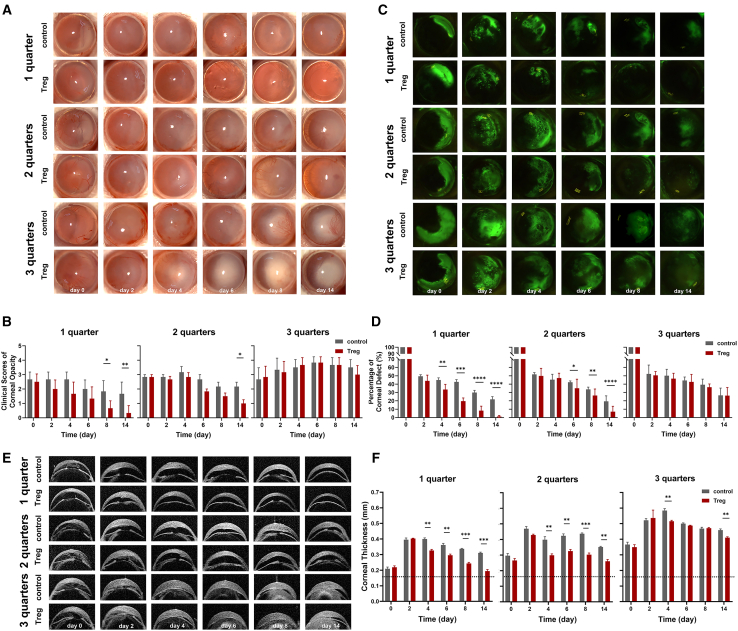
Subconjunctival injection of Tregs was effective in treating mild and moderate LSCD In the corneal limbus, filter paper rings with a variety of central angles (90, 180, or 270°) were utilized to deliver alkali burn to different sizes of limbal area (1, 2, or 3 quarters) so that animal models of mild, moderate, and severe LSCD were made. (A and B) Corneal opacity was shown in representative bright-field corneal images photographed by slit-lamp microscope and scored in the summary graphs. (C and D) Corneal epithelial defect was shown in representative fluorescein staining images. The areas of defect were normalized to day 0 as percentages of initial defect and summarized in the bar graphs. (E and F) Representative optical coherence tomography (OCT) images displayed corneal thickness at indicated time points after injury. The horizontal dotted line shows the thickness of the corneas of normal mice. The summary of corneal thickness measurements obtained via OCT is presented in the bar graphs. Data are presented as the mean ± SEM; ∗*p* < 0.05, ∗∗*p* < 0.01, ∗∗∗*p* < 0.001, ∗∗∗∗*p* < 0.0001; *n* = 6.

We employed a mouse model of alkali burn-induced limbus injury to test the *in vivo* efficacy of subconjunctival injection of Tregs. 1-, 2-, or 3-quarter of limbus ring (sparing cornea and conjunctiva) were burned to induce mild (1-quarter alkali burn), moderate (2-quarter alkali burn), and severe (3-quarter alkali burn) LSCD, as described previously.^32^ Bright-field corneal imaging demonstrated that subconjunctival injection of Tregs started to reduce corneal opacity scores in mild LSCD as early as day 2 after injury and led to significantly lower scores by day 8 and day 14 than the control treatment, with corneal transparency almost completely restored by the end of follow up (day 14); the treatment also progressively decreased opacity in moderate LSCD starting from day 6 throughout to day 14, by which the scores in Treg group were significantly lower than the control treatment; however, Treg injection did not lead to any significant improvement in corneal opacity in severe LSCD as compared to the control treatment (Figures 1A and 1B). In addition, although there was no direct burn on central cornea, damage to limbus resulted in adjacent corneal epithelial defect in all LSCD models, demonstrated by the corneal fluorescein staining. Compared to the control treatment, Treg treatment resulted in significantly smaller wound areas from day 4 to day 14 in mild LSCD and from day 6 to day 14 in moderate LSCD. In contrast, there were no significant differences in wound areas between control and Treg groups in severe LSCD during the observation period (Figures 1C and 1D). Furthermore, we used optical coherence tomography (OCT) imaging to evaluate corneal edema by measuring central corneal thickness (CCT). We found that all LSCD models experienced a fast and persistent increase in CCT, with the highest CCT occurring in the severe model. Treg treatment led to a significant reduction of CCT from day 4 through day 14 in both mild and moderate LSCD models compared to the control treatment. In severe LSCD, Treg treatment led to significantly lower thickness than control treatment only on days 4 and 14, but the thickness after Treg treatment was still much higher than normal (Figures 1E and 1F).

These findings demonstrate that local Treg injection can effectively suppress the development of mild or moderate limbus injury (no more than 1/2 limbus)-associated corneal edema and epithelial defect. However, in cases of severe LSCD involving 3 quarters limbus injury, such abilities of exogenous Tregs treatment were diminished, suggesting that the tissue repairing abilities of Treg treatment are associated with the existence of residual healthy LSCs.

### Co-culturing with Tregs promotes the migratory capacity of LSCs

To elucidate the underlying mechanism by which Treg treatment ameliorated alkali burn-induced LSCD, a series of *in vitro* experiments were conducted using the co-culture of primary human LSCs with human Tregs derived from peripheral blood mononuclear cells (PBMCs) of healthy donors. The purity of sorted CD4^+^CD25^+^CD127^dim^ Tregs^33^^,^^34^ was confirmed as >90% by flow cytometry analysis (Figure S1A). These cells were utilized for co-culturing and subsequent downstream analysis. In the co-culture, human LSCs were adhered to the plates as larger, more elongated, or spindle-shaped cells, while Tregs were non-attached, smaller, round-shaped cells (Figure S1B).

**Figure 2:**
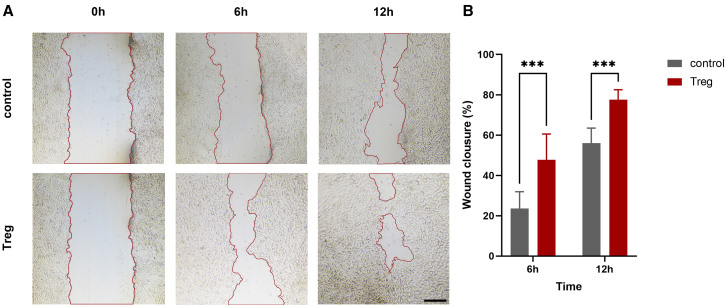
Co-culturing with Tregs promotes the migratory capacity of LSCs (A) Representative images depicting the scratch assay from both groups were captured at 0, 6, and 12 h. The dotted lines delineate the interfaces or cell fronts. Scale bar, 100 μm. (B) The percentages of wound healing area were analyzed as wound closure rate and subjected to comparative assessment. Data are presented as the mean ± SEM; ∗*p* < 0.05, ∗∗*p* < 0.01, ∗∗∗*p* < 0.001; *n* = 6.

Scratch assays were conducted to characterize the migratory capacity of LSCs after co-culture with Tregs. Observations were made under a microscope at 0 h, 6 h, and 12 h post-scratching. At 6 h post-scratch, LSCs gradually migrated toward the central area of the scratch, with a notably faster migration rate in the Treg group. By 12 h post-scratch, the gap in Treg-treated LSCs was partially closed, which was not observed in the control group (without pre-coculture with Tregs) (Figure 2A). The statistical results show significantly more wound closure in Treg-treated LSCs at 6 h and 12 h (Figure 2B). These findings suggest that Tregs have the potential to promote the migration of LSCs.

### Co-culturing with Tregs preserves the stemness while suppressing the differentiation potential of LSCs

**Figure 3:**
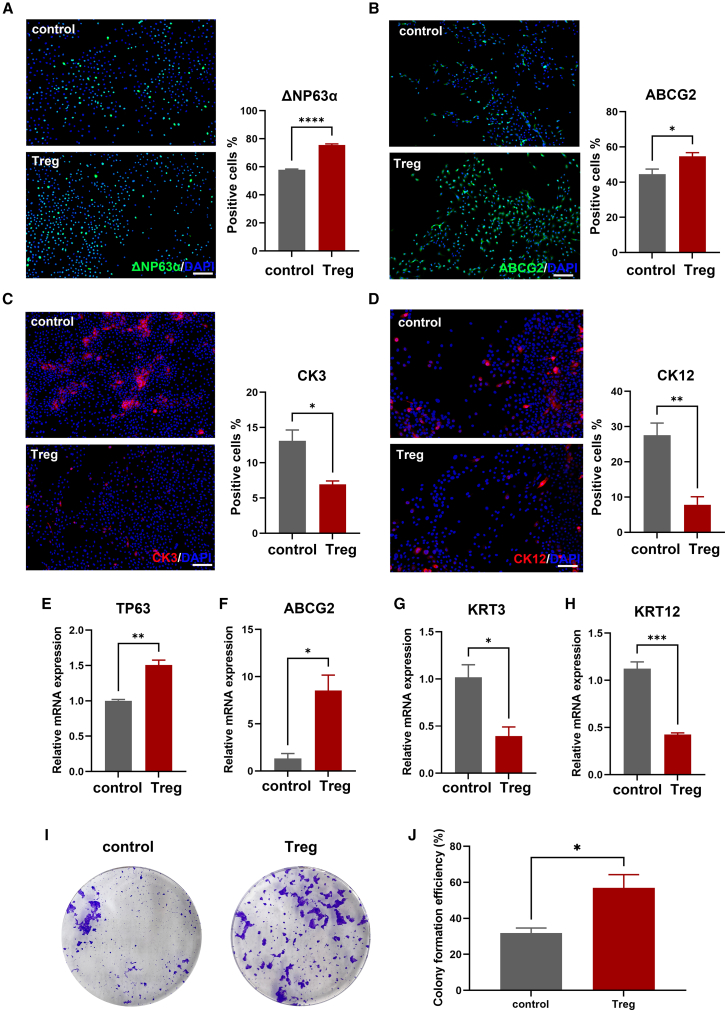
Co-culturing with Tregs preserves the stemness characteristics and inhibits the differentiation of LSCs (A–D) Immunofluorescence staining images of ΔNP63α, ABCG2, CK3, and CK12 of LSCs at 24 h post co-culture are presented. The positive cell count chart is located to the right of the fluorescence images. Nuclei were counterstained with DAPI. Scale bars, 100 μm. (E–H) The relative mRNA expression levels of TP63, ABCG2, KRT3, and KRT12 in LSCs following a 24-h co-culture with or without Tregs were evaluated. (I and J) Representative images depicting formation of LSC colonies in each group were captured 12 days post-co-culture. Data are presented as the mean ± SEM; ∗*p* < 0.05, ∗∗*p* < 0.01, ∗∗∗*p* < 0.001, ∗∗∗∗*p* < 0.0001; *n* = 3.

We next examined how the inherent characteristics related to stemness and differentiation in LSCs were influenced after being co-cultured with Tregs. Immunofluorescence staining showed that while Treg co-culture preserved the inherent stemness of LSCs, it did hinder their transition into corneal epithelial cells. After co-culturing with Treg cells, there was a significant increase in the proportion of LSC cells expressing the stemness markers ΔNP63α, ABCG2^4^ (Figures 3A and 3B), as well as CK14 and CK19 (Figure S2). Conversely, the percentages of cells positive for corneal epithelial differentiation markers, CK3 or CK12,^35^^,^^36^ were significantly decreased (Figures 3C and 3D). Further quantification using reverse transcription qPCR (RT-qPCR) analysis showed consistent changes as observed by the immunofluorescence staining, i.e., increased mRNA expression of genes TP63 and ABCG2 (Figures 3E and 3F) and decreased mRNA expression of genes KRT3 and KRT12 (Figures 3G and 3H). Given that colony-forming ability stands as a crucial characteristic of stem cells, we proceeded to compare the colony-forming efficiency (CFE) among two groups. The figures illustrated the quantity of clones, displaying visibly more clones in the Treg group (Figure 3I). Statistical analysis of CFE also indicated a trend toward higher CFE in the Treg group (Figure 3J).

### Tregs substantially enhance the proliferative capacities of LSCs

**Figure 4:**
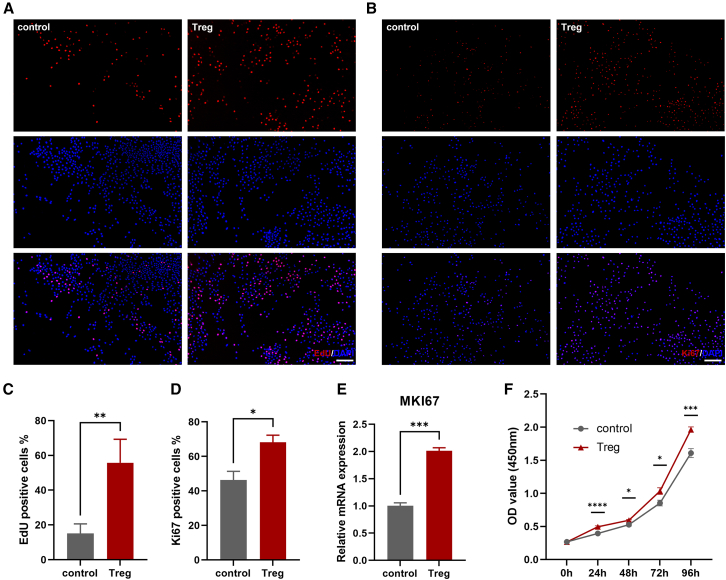
Tregs exert a substantial stimulatory effect on LSC proliferation (A) Representative immunofluorescence images of EdU incorporation (red) of LSCs at 24 h post co-culture are presented for each group. Nuclei were counterstained with DAPI. Scale bar, 100 μm. (B) Representative immunofluorescence images of Ki67 of LSCs (red) at 24 h post co-culture are presented for each group. Nuclei were counterstained with DAPI. Scale bar, 100 μm. (C) The percentage of EdU-positive cells was analyzed based on the immunofluorescence staining results. (D) The percentage of Ki67-positive cells was quantified for analysis. (E) The relative mRNA expression levels of the gene MKI67 in LSCs following a 24-h co-culture with or without Tregs were evaluated. (F) The proliferation of each cell group was assessed using the CCK-8 assay, and the relative absorbance was demonstrated at 450 nm. Data are presented as the mean ± SEM; ∗*p* < 0.05, ∗∗*p* < 0.01, ∗∗∗*p* < 0.001; *n* = 3.

For a more comprehensive understanding of the impact of Tregs on the proliferation of LSCs, a series of assays were performed. First, we conducted EdU incorporation experiments to evaluate the cells in the S-phase.^37^ The results showed significantly higher proportions of S-phase cells in the Treg co-culture group than the control group (Figures 4A and 4C). Additionally, the nuclear staining of the proliferation marker Ki67 was analyzed to evaluate cell proliferation. Results showed that LSCs co-cultured with Tregs exhibited a significantly higher Ki67-positive rate (Figures 4B and 4D). The mRNA expression levels of Ki67 in LSCs were consistent with the immunofluorescence findings, further confirming the increased proliferative capacity of LSCs co-cultured with Tregs (Figure 4E). Last, we employed the CCK-8 assay to measure the proliferation rates of LSCs. LSCs co-cultured with Tregs exhibited significantly higher proliferation after 24 h, which was progressively increased and remained substantially higher than LSCs without co-culture with Tregs through 96 h (Figure 4F). Collectively, these results demonstrate that co-culturing with Tregs significantly enhances LSC proliferation.

### Tregs regulate multiple genes associated with LSC function

**Figure 5:**
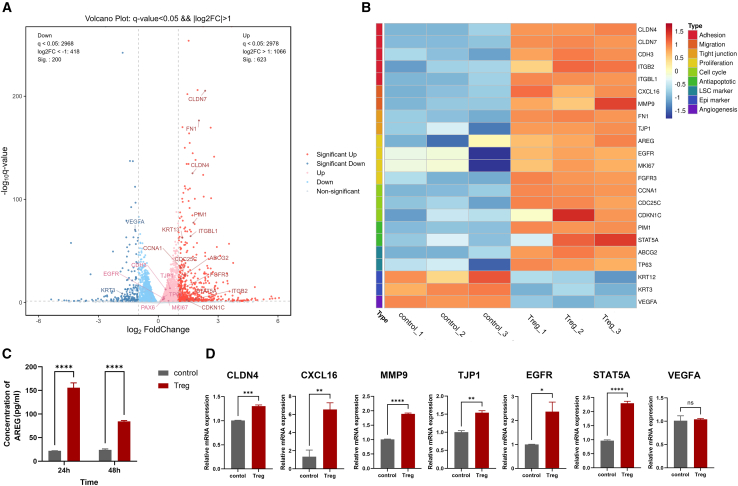
RNA-seq analysis and confirmation experiments reveal the regulation of LSCs functional genes by Tregs (A) The volcano map depicted the distribution of DEGs, where red and blue dots represent genes significantly upregulated or downregulated in the Treg group, respectively (fold change >2 and *q* value < 0.05). (B) The heatmap illustrated differentially expressed functional genes closely associated with the mechanism related to adhesion, migration, tight junction, proliferation, cell cycle, antiapoptotic, LSC markers, epithelial markers, and angiogenesis of LSCs. (C) The protein content of AREG in the supernatant after 24 or 48 h of co-culturing LSCs with or without Tregs was assessed by ELISA. (D) The relative mRNA expression levels of several genes related to adhesion, migration, tight junctions, proliferation, anti-apoptotic processes, and angiogenesis (CLDN4, CXCL16, MMP9, TJP1, EGFR, STAT5A, and VEGFA) in LSCs following a 24-h co-culture with or without Tregs were evaluated. Data are presented as the mean ± SEM; ∗*p* < 0.05, ∗∗*p* < 0.01, ∗∗∗*p* < 0.001,∗∗∗∗*p* < 0.0001 ns not significant; *n* = 3.

To comprehensively explore the differential gene expression of LSCs before and after co-culturing with Tregs, the whole transcriptome RNA-seq was conducted on LSCs. An overview of the differentially expressed genes (DEGs) is depicted in the volcano plot. As depicted in the volcano plot, in comparison to the control group, LSCs co-cultured with Treg exhibited a significant upregulation in the expression of 623 genes and a significant downregulation in the expression of 200 genes (Figure 5A). The heatmap demonstrated differential gene expression related to adhesion, migration, proliferation, cell cycle, and apoptosis in LSCs. As depicted in the figure, co-culturing with Tregs led to upregulation of adhesion-related genes CLDN4, CLDN7, CDH3, ITBG2, ITGBL1, migration-related genes CXCL16, MMP9, tight junction-related genes FN1, TJP1, proliferation-associated genes EGFR, MKI67, FGFR3; cell cycle-associated genes CCNA1, CDC25C, CDKN1C; anti-apoptotic genes PIM1, STAT5A. Also observed were upregulated genes related to stemness, such as ABCG2, and TP63, while downregulated genes included differentiation-related genes like KRT12, KRT3, and angiogenesis-related genes like VEGFA (Figure 5B). The findings were subsequently validated by ELISA analysis of AREG in the supernatant (Figure 5C) and RT-qPCR (quantitative reverse transcription PCR) analyses of multiple molecules in LSCs (Figure 5D), highlighting the role of Tregs in regulating genes facilitating migration, proliferation, and maintenance of stemness in LSCs.

### The effect of Treg on LSCD recovery relies on the expression of AREG

**Figure 6:**
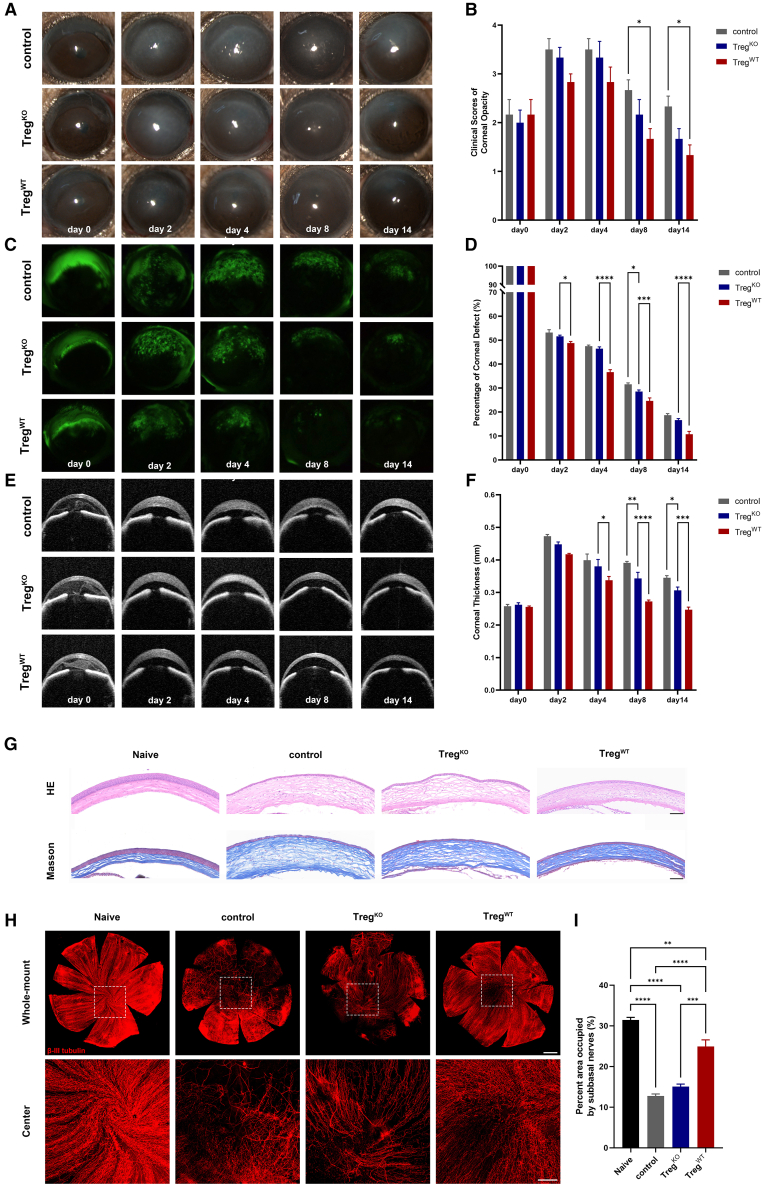
Subconjunctival injection of AREG^−/−^ Treg exhibited less efficacy in treating moderate LSCD than WT Treg Moderate LSCD mouse models were made. Treg^KO^ and Treg^WT^ were isolated from spleens of AREG^−/−^(KO) and wild-type (WT) mice, respectively, and injected subconjunctivally into moderate LSCD mice at equal cell concentrations. The control group received an equivalent volume of PBS injection. (A and B) Corneal opacity was shown in representative bright-field images photographed by slit-lamp microscope and scored in the summary graphs. (C and D) Corneal epithelial defect was shown in representative fluorescein staining images. The areas of defect were normalized to day 0 as percentages of initial defect and summarized in the graph beside. (E and F) Representative optical coherence tomography (OCT) images displayed corneal thickness after injury. The summary of corneal thickness measurements is presented in the bar graphs. (G) Representative hematoxylin and eosin (HE) and Masson’s Goldner (Masson) staining images of central corneas. Scale bar, 100 μm. (H) Representative images of the whole cornea (upper) and central cornea with spiral-shaped subbasal corneal nerves (lower) in different groups (β III tubulin, red). Scale bars, 500 μm (upper) or 200 μm (lower). (I) Statistical analysis of the nerve density in the central zone of the cornea. Data are presented as the mean ± SEM; ∗*p* < 0.05, ∗∗*p* < 0.01, ∗∗∗*p* < 0.001, ∗∗∗∗*p* < 0.0001; *n* = 6(B, D, and F) and 3(I).

A previous study has demonstrated that administration of anti-AREG antibodies attenuated the reparative effect of Tregs.^31^ In this study, we generated AREG^−/−^ mice and isolated AREG^−/−^ Tregs (Treg^KO^) from their spleens. Treg^KO^, Treg^WT^, or PBS were then subconjunctivally injected to mice with moderate LSCD. Compared to Treg^WT^, mice treated with Treg^KO^ exhibited slower restoration of corneal transparency, with clinical scores on day14 similar to those of mice treated with Treg^WT^ on day 8 post-injury (Figures 6A and 6B). Similarly, the recovery rate of epithelial defect in the Treg^KO^ group was also slower than that in the Treg^WT^ group. The percentage of epithelial defective area was significantly higher in Treg^KO^ group than in Treg^WT^ group as early as day 2 and throughout to the end of observation (day 14) (Figures 6C and 6D). Consistently, OCT results indicated significantly decreased capacity of Treg^KO^ than Treg^WT^ in reducing corneal thickness, with significant differences observed from day 4 to day 14 (Figures 6E and 6F). However, overall, the recovery rate of mice in the Treg^KO^ group still remained faster than the PBS group. After the clinical follow-up period, mice were euthanized, and their eyes were subjected to histological examination and corneal nerve staining. Hematoxylin and eosin (HE) staining and Masson’s trichrome staining (Masson) showed that both the control PBS group and Treg^KO^ group displayed poor histomorphology, characterized by abnormal cell morphology and arrangement of corneal epithelium, along with loose and disordered corneal stromal fiber arrangement. In contrast, corneas in Treg^WT^-treated group exhibited thicker epithelium and denser stroma, close to normal corneas (Figure 6G). The corneas of each group were subjected to β III tubulin staining to visualize the distribution of nerve fibers (Figure 6H). Analysis of representative images revealed that Treg^WT^ treatment significantly prevented the loss of corneal nerve fibers in LSCD. In contrast, Treg^KO^ did not exhibit such capacities (Figure 6I).

## Discussion

LSCD is characterized by the loss or dysfunction of LSCs, and it represents a challenging ocular condition. LSCD can result from various etiologies, from genetic disorders to environmental influences like chemical burns, excessive contact lens wear, and persistent inflammation.^38^ The pathological consequences of LSCD encapsulate a spectrum of ocular manifestations, including persistent epithelial defects, corneal conjunctivalization, neovascularization, and scarring, culminating in visual impairment or blindness.^39^ The therapeutic approach for LSCD has evolved, yet remains fraught with challenges. Limbal tissue or cell transplantation is currently the mainstay for treating LSCD and can be classified based on the origin of stem cells (e.g., autologous and allogeneic), the type of stem cell graft (e.g., limbal and non-limbal), and whether *ex vivo* expansion of stem cells in culture is employed.^40^ However, scarcity of suitable donor tissues and risk of immune rejection, especially in allogeneic transplants,^41^^,^^42^ are the main hurdles. Simple limbal epithelial transplantation (SLET), a newer modality, has emerged as a promising option for mild LSCD. SLET offers a decreased risk of iatrogenic LSCD in the healthy cornea during tissue harvesting, direct transfer without the necessity for cell culture, and the ability to conduct biopsy harvest and transplantation in a single procedure. Despite its advantages, SLET’s applicability to severe cases of LSCD remains constrained, underscoring the imperative need for novel therapeutic strategies.^43^^,^^44^ The present study demonstrates that Tregs enhance LSC function and suppress the development of LSCD-associated corneal pathologies.

Recently, an increasing body of research has suggested regulation of stem cell functions by Tregs, a highly intricate subset of lymphocytes with specialized immunosuppressive function.^45^ Specifically, Tregs have been shown to facilitate tissue repair and maintain tissue homeostasis^46^ beyond their roles in immune surveillance. In a recent study, Burzyn et al. identified the involvement of Tregs in muscle regeneration through local immunomodulation and the expression of AREG.^47^ Additionally, Arpaia et al. reported the tissue-protective functions of AREG-expressing Tregs independent of T cell receptor (TCR) signaling in the context of influenza-infected lungs.^29^ Dombrowski et al. revealed a crucial regenerative role of Tregs in accelerating oligodendrocyte differentiation and (re)myelination through CCN3.^28^ Wang et al. suggested that activated Tregs promote neural stem cell (NSC) proliferation in both normal and ischemic mice within the subventricular zone.^48^ Notably, Ali et al. showed that Tregs in skin hair follicles co-localized with and supported the function of hair follicle stem cells (HFSCs), facilitated by Jag1 expression on Tregs.^25^

In our previous study in a central corneal injury model with limbus spared, Treg injection was proved highly effective.^31^ However, in clinical practice, treatment outcomes are closely correlated with the degree of limbal damage and LSCD. The more severe the LSCD, the poorer the prognosis. Therefore, our current study has explored the therapeutic effect of Tregs in an LSCD model, complementing previous research findings and further elucidating the effects of Tregs in promoting LSC function for the first time to our knowledge. Following an equivalent number of Tregs subconjunctival injections in LSCD animal models, we observed that Tregs facilitated the repair of alkali burn-induced limbus injury by reducing corneal edema and promoting epithelial healing in the mild and moderate models. However, as the injury involved a larger (3/4) limbal area, the efficacy of Tregs was diminished. These data suggest that the function of Tregs in ameliorating LSCD is probably dependent on the residual quantity of LSCs in the limbus. To further explore the underlying mechanisms of action of Tregs, we performed cell co-culture experiments using human Tregs and LSCs. We found that LSCs co-cultured with Tregs demonstrated a notable accelerated cell migration, better preserved stemness markers, higher colony-forming ability and decreased expression of mature corneal epithelial markers, along with the heightened capabilities of proliferation. Furthermore, RNA-seq data showed similarly altered expressions of relevant genes involved in migration, stemness, differentiation, and proliferation of LSCs and additionally provided insight into the potential impact of Tregs on LSCs in promoting antiapoptotic gene expression while suppressing angiogenesis-related gene expression.

Furthermore, compared to injection of WT Tregs, injection of AREG^−/−^ Tregs to LSCD mice exhibited decreased effects in alleviating LSCD, but there was still improvement relative to the control group. *In vitro* experiments, the direct addition of recombinant AREG to LSCs cultures in the absence of Tregs promoted the proliferation and migration of LSCs but had no significant effect on maintaining stemness (Figure S3). While our findings suggest that AREG contributes to LSC migration, the specific molecular mechanisms remain to be fully elucidated. Future studies should investigate whether AREG regulates cytoskeletal remodeling via key signaling proteins^49^ (e.g., FAK, paxillin, Cdc42, Rac1, and RhoA) and whether its effects are dependent on actin polymerization.

Our previous results indicated that blocking AREG partially impaired the regenerative function of Tregs.^31^ Therefore, we believe AREG serves as a key factor in Treg-mediated repair of LSCD, likely playing a supportive role alongside other Treg-secreted factors. AREG is an epidermal growth factor (EGF) family member known to promote wound healing and regeneration of different tissues. In the previous research, a loss-of-function experiment involving anti-AREG antibody treatment combined with Tregs demonstrated a diminished anti-inflammatory capacity of Tregs.^31^ In muscle repair, Tregs have been demonstrated to be located close to regenerating fibers and express AREG to enhanced satellite cell, a muscle stem cell responsible for the repair of injured muscle.^47^ Tregs in the brain have been reported to promote neuronal stem cell proliferation through effector molecules including AREG, IL-10, BNDF, and galectin 1.^50^^,^^51^ During influenza virus infection, AREG derived from Treg cells has been shown to stimulates the activation and survival of EGFR^+^Collagen14^+^ lung mesenchymal cells, which act as signaling relay intermediates to facilitate effective alveolar regeneration.^52^ Our previous results indicate that the EGFR signaling axis is likely a critical mediator of the observed outcomes.^31^ In this research, we identified the enrichment of DEGs in the nuclear factor kappa B (NF-κB) signaling pathway through RNA-seq analysis (Figure S4). NF-κB has been demonstrated to play a dual role by inducing the migration and proliferation of human MSCs,^53^ as well as promoting the proliferation of corneal epithelial progenitors.^46^ Previous studies have also reported that AREG acts through the EGFR/ERK/NF-κB signaling pathway during epithelial-mesenchymal transition, in which cell migration and proliferation was involved.^54^ However, the precise roles of EGFR, the NF-κB pathway, and their potential interactions with LSCs remain to be fully elucidated.

Interestingly, after co-culturing Tregs with LSCs, we observed that once the Tregs were removed and the LSCs were cultured and passaged independently, there was no noticeable difference compared to the control group. Also, the natural senescence of LSCs, which generally occurs after 5–6 passages in culture, cannot be delayed by Tregs (data not shown). This suggests that the effects of Tregs on LSCs are not long-lasting and require the continuous presence of Tregs, and Tregs cannot completely reverse the inevitable senescence fate of LSCs.

Given the inadequate effect of Treg alone treatment in severe LSCD demonstrated by this study, future research might focus on two areas to improve the therapeutic efficacy of Tregs-based approach. First, efforts should be directed toward enhancing the functionality of Tregs. This could involve modulating the expression or activity of key cytokines and growth factors, such as AREG and IL-10 known for their roles in tissue regeneration and inflammatory suppression. Secondly, it is worth exploring the potential of combining Treg therapy with stem cell injections. Future research should focus on optimizing *in vivo* experiments to confirm the role of Treg in promoting LSC function. This could include advanced staining techniques to better visualize LSC proliferation, conducting LSC transplantation in LSCD animal models to determine whether primed LSCs (after co-culture with Tregs) exhibit enhanced tissue repair potentials, as well as investigating the molecular mechanisms underlying Treg-mediated regulation of LSCs. Additionally, exploring the long-term effects of Treg injection on limbus regeneration and assessing its potential therapeutic applications would provide valuable insights. We hope to provide a more comprehensive approach to overcoming the challenges posed by low numbers of residual LSCs in severe cases of LSCD.

In summary, this study demonstrates the therapeutic promise of Tregs in treating LSCD by enhancing the function of residual LSCs, with Treg-derived AREG likely playing a pivotal role. By advancing our understanding of the mechanisms underlying the interactions between Tregs and LSCs and refining therapeutic approaches, we can strive toward more effective and comprehensive strategies.

### Limitations of the study

First, the relatively short observation period could be the reason for the absence of corneal neovascularization, fibrosis or conjunctivalization in the LSCD model. Second, this study did not examine the proliferation or migration of residual LSCs after Tregs injection *in vivo*. LSCs co-cultured *in vitro* should be transplanted into LSCD animal models to verify their efficacy. Third, further investigation into the mechanisms or signaling pathways underlying the interaction between Tregs and LSCs, especially those involving the tissue repair function of AREG, is needed.

## Resource availability

### Lead contact

Requests for further information and resources should be directed to and will be fulfilled by the lead contact, Chunyi Shao (wujunshabu@126.com).

### Materials availability

This study did not generate new unique reagents.

### Data and code availability

Raw RNA-seq data generated during this study are deposited in GEO and publicly available as of the date of publication. Accession number is GSE291731. Other data reported in this paper will be shared by the lead contact upon request.

This paper does not report original code.

Any additional information required to reanalyze the data reported in this paper is available from the lead contact upon request.

## Acknowledgments

This research was supported by the 10.13039/501100001809National Natural Science Foundation of China (82471040, 82471039, and 82271041); Shanghai JiaoTong University School of Medicine Two-hundred Talent (20191914); the 10.13039/501100012247Program of Shanghai Academic/Technology Research Leader (22XD1401800); 10.13039/100017950Shanghai Municipal Health Commission Clinical Research Special Program (Excellence Project) (20254Z0008).

We would like to express our sincere gratitude to the State Key Laboratory of Eye Health and Shanghai Key Laboratory of Orbital Diseases and Ocular Oncology for providing the research platform. Graphical abstract was created with BioRender.com.

## Author contributions

F.F. and T.A. designed the experiments, analyzed the data, and prepared the manuscript; J.C. conducted the experiments and analyzed the data; S.L. and T.Z. conducted the experiments; L.C. and Y.F. assisted with the study design and reviewed the manuscript; C.S. secured funding, designed the study and reviewed the manuscript; all authors have read and approved the final version of the manuscript.

## Declaration of interests

The authors declare no competing interests.

## STAR★Methods

### Key resources table

**Table:** 

REAGENT or RESOURCE	SOURCE	IDENTIFIER
**Antibodies**		

anti-Ki67	abcam	ab15580
anti-beta III Tubulin	abcam	ab52623
anti-CK14	abcam	ab7800
anti-CK19	Invitrogen	MA5-15884
anti-CD4-BV421	Invitrogen	404-0042-82
anti-CD25-PE	Invitrogen	12-0251-82
anti-CD127-FITC	Invitrogen	11-1271-82
anti-ABCG2	santa cruz	sc-377176
anti-ΔNp63α	santa cruz	sc-25268
anti-CK3	santa cruz	sc-80000
anti-CK12	santa cruz	sc-515882

**Biological samples**		

Human limbus rim	Shanghai Ninth People’s Hospital	N/A
Human peripheral blood	Shanghai Ninth People’s Hospital	N/A

**Chemicals, peptides, and recombinant proteins**		

Human AREG	MCE	HY-P70578
Human EGF	Sigma-aldrich	324831
Y-27632	Sigma-aldrich	SCM075

**Critical commercial assays**		

Cell Counting Kit 8 (WST-8/CCK8)	abcam	ab228554
Click-iT™ cell proliferation kit	Invitrogen	C10339
Rneasy Mini Kit	Qiagen	74104
PrimeScript™ RT Reagent Kit	TaKaRa	RR037B
CD4^+^CD25^+^ Regulatory T cell Isolation Kit, mouse	Miltenyi	130-091-041

**Deposited data**		

Raw and analyzed RNA-seq data	This paper	GSE291731

**Experimental models: Organisms/strains**		

Mouse: BALB/c and C57BL/6	Shanghai JieSiJie Laboratory Animal Co.,Ltd	N/A
Mouse: AREG^−/−^ mouse on C57BL/6 background	Shanghai Model Organisms Center	N/A

**Oligonucleotides**		

Primer sequences for RT-qPCR	This paper	Table S1

**Software and algorithms**		

ImageJ 1.52v	ImageJ	N/A
GraphPad Prism 9	GraphPad	N/A
R software (4.1.2)	R	N/A

### Experimental model and study participant details

#### Human participants

Anticoagulated human peripheral blood (50mL) was collected from healthy donors after informed consent at the Department of Ophthalmology, Ninth People’s Hospital, affiliated with Shanghai Jiao Tong University School of Medicine. Detailed participant characteristics were shown in Table S2. Human corneal rims derived from the donor corneas for transplantation were obtained from the Department of Ophthalmology, Ninth People’s Hospital, affiliated with Shanghai Jiao Tong University School of Medicine. All participants were treated in accordance with the Declaration of Helsinki. All procedures were approved by the ethics committee of Shanghai Ninth People’s Hospital (Ethics approval number: SH9H-2020-T70-2).

#### Animal models

The animal studies received ethical approval from the Medical Ethics Committee of Ninth People’s Hospital, Shanghai Jiao Tong University School of Medicine (Ethics approval number: SH9H-2020-A177-1), adhering to the guidelines of the Chinese Animal Administration and the Association for Research in Vision and Ophthalmology guidelines for animal use in ophthalmic and vision research. Female BALB/c or C57BL/6 mice, aged 6–8 weeks, were purchased from Shanghai JieSiJie Laboratory Animal Co.,Ltd. Animals were housed in a sterile environment from the Animal Center of Ninth People’s Hospital, Shanghai Jiao Tong University School of Medicine. Mice underwent anesthesia and received an ocular application of oxybuprocaine hydrochloride eye drops (Santen Pharmaceutical, Kita-ku, Osaka, Japan). Whatman III filter paper rings with diverse central angles (90, 180, or 270°) presoaked in 1 N NaOH (Sigma-Aldrich) were positioned within the limbal region, ensuring no contact with the central cornea or conjunctiva for 30 s to induce varying degrees of limbus damage, as described previously.^32^ Subsequently, the exposed eye underwent thorough rinsing with a minimum of 15mL of PBS (Sigma-Aldrich, St. Louis, MO, USA) for 1 min. Thus, we established alkali burn-induced mild (1-quarter Alkali Burn), moderate (2-quarter Alkali Burn), and severe (3-quarter Alkali Burn) LSCD.

### Method details

#### Animal treatment and clinical evaluation

Mouse CD4^+^CD25^+^ Tregs (mTregs) were isolated from spleens of AREG^−/−^ mice (Treg^KO^ on C57BL/6 background, Shanghai Model Organisms Center) and wide-type C57BL/6 or BALB/c mice (Treg^WT^) using MACS sorting according to protocols of mouse Treg cell isolation kit (Miltenyi Biotec, Bergisch-Gladbach, Germany). The purity of mTregs among sorted cells was >90%, as examined by flow cytometric analysis.

The mice with mild, moderate, or severe LSCD were randomly divided into control and Treg groups, respectively. The control group received a subconjunctival injection of 10μL PBS, whereas the Treg group received an injection of 1×10^5^ mTregs resuspended in 10μL PBS using a 33-gauge metal needle and a 50-μL syringe (Hamilton, Reno, NV). All subconjunctival injections were administered proximal to the uninjured quadrant of the cornea.

Clinical assessments were performed for each group in a blinded fashion on days 0, 2, 4, 6, 8, and 14 post-injury. The corneal opacity was first evaluated using a slit-lamp microscope (SL-D7, Topcon, Tokyo, Japan) and scored in a range from 0 to 4: 0 = completely transparent; 1 = slightly cloudy, with iris and pupils easily visible; 2 = moderately cloudy, with discernible iris and pupils; 3 = highly cloudy, with minimal visibility of iris and pupils; and 4 = fully cloudy, obscuring the iris and/or pupils entirely.^55^ Subsequently, corneal fluorescein staining with 2% fluorescein sodium (Sigma-Aldrich) was conducted, and cobalt blue light was used to photograph and evaluate corneal epithelial defects. Additionally, the CCT was measured using OCT (Heidelberg Engineering, Germany), and an average of three readings were recorded for analysis.

#### Isolation and expansion of human Tregs

Human PBMCs were isolated from anticoagulated peripheral blood by Ficoll-Paque gradient centrifugation density (GE Healthcare) according to the manufacturer’s instructions. The viability of PBMCs was determined by trypan blue exclusion method on a hemacytometer. Human PBMCs (viability>90%) were stained with anti-CD4-VioBlue (Miltenyi Biotec) and anti-CD25-PE (Miltenyi Biotec) for flow cytometry. CD4^+^CD25^+^ Tregs isolation was performed by flow cytometric sorting using MACSQuant Tyto Sorter (Miltenyi Biotec) in MACSQuant Tyto Running Buffer (Miltenyi Biotec). The pre- and post-sorted populations were analyzed and counted by flow cytometry using the MACSQuant Analyzer.

The freshly sorted human CD4^+^CD25^+^ Tregs (hTregs) were seeded at a density of 5×10^5^ cells/ml in a 24-well or 48-well culture plate in TexMACS GMP Medium (Miltenyi Biotec) containing 5% heat-inactivated human AB serum (Sigma), 50 U/mL penicillin and streptomycin antibiotics (Gibco), and 500 IU/mL human IL-2 (Miltenyi Biotec). At day 0, Tregs were activated by adding anti-CD3 and CD28 beads (T cell TransAct human, Miltenyi Biotec) at 10 μL/mL. Cell cultures were gently mixed and incubated at 37°C with 5% CO_2_ for 3 days. At day 3, cells were washed with fresh supplemented medium and the cell suspension was split every 2 days and adjusted for a cell number of 3–5 × 10^5^ cells/ml by partial media replacement (50%). Cells were counted by the trypan blue exclusion method on a hemacytometer. The purity of hTregs was determined by flow cytometric analysis using anti-human monoclonal antibodies (mAbs): CD4-BV421(Invitrogen), CD25-PE(Invitrogen), CD127-FITC(Invitrogen). Appropriate isotype controls and fluorescence minus one control samples were used and flow cytometry was performed on the BD LSR Fortessa X-20(BD Bioscience), and data were analyzed using FlowJo software.

#### Human Limbal stem cells isolation and culture

Human cornea rims were rinsed and soaked several times in PBS containing 50 U/mL penicillin and streptomycin antibiotics (Gibco) and digested with Dispase II (Sigma, 5 mg/mL) in DMEM/F12 medium (Sigma-Aldrich) at 4°C for 8h. Epithelial layer was peeled off and the epithelium sheet was dissociated into single cells using 0.05% trypsin (Invitrogen) for 10 min at 37°C. The cells were then seeded on a 10 cm culture dish and cultured with supplemented Dulbecco’s modified Eagle’s medium (DMEM)/F12 (Invitrogen) containing 10% fetal bovine serum, 5 μg/mL insulin, 5 μg/mL transferrin, 5 ng/mL selenium, 0.5 μg/mL hydrocortisone, 0.5% dimethyl sulfoxide, 2 ng/mL human EGF, 20 μM Y-27632 (Sigma), and 50 U/mL penicillin and streptomycin antibiotics (Gibco). After 24 h, the non-adherent cells were removed by PBS. When the adherent cells reached a confluency of approximately 80–90%, the cells were digested into single cells by trypsin and cultured. The first to third passages of LSC were used for further experiments.

#### Tregs and Limbal stem cells co-culture

Human LSCs were counted and seeded in 24-well or 6-well plates at a density of 25000 cells/cm^2^ 6 h before experiment for attachment. Human Tregs were directly placed into the LSC wells in a ratio of LSC: hTreg as 1:1, and cultured together for 24 h. Before analysis, hTregs were removed by PBS washing, and LSCs were then harvested by enzymatic digestion with 0.25% trypsin-EDTA at 37°C for 5 min, and prepared into single cell suspensions.

#### Cell Count Kit-8 (CCK-8) assay

The number of viable LSCs was indirectly determined using Cell Count Kit-8 assay (Abcam) according to the manufacturer’s instructions. Briefly, LSCs of second passages were seeded in 96-well plates (2000 cells/well) for 6 h adherence, then co-culture with or without hTregs. During the *in vitro* co-culture process, we observed that Tregs gradually undergo cell death in the LSC medium after 48 h. Therefore, we refreshed the culture medium (and replaced fresh Tregs) in all experimental groups every 48 h. Before 10 μL of the CCK-8 solution was added, each well was washed with PBS 3 times to remove Tregs. Then the absorbance at 450 nm was measured by a Microplate Reader (Thermo Fisher Scientific) after 2 h’ incubation.

#### Scratch assay

LSCs were seeded in 24-well culture plates at a cell density of 8 × 10^4^/well and cultured in a supplemented culture medium until a confluent cell monolayer formed. A scratch wound was made in the center of each well with a 200-μL pipet tip. Tregs cells were added into the LSC wells, and co-cultured for 12 h. 10 mg/mL mitomycin C was added to the solution to inhibit cell proliferation. Before imaging, the liquid in each well is aspirated, followed by gentle washing with PBS 3 times. Images were acquired at the same position using a bright-field microscope to record cell migration. The wound area was calculated using the ImageJ software, while the migration rate can be expressed as the percentage of wound closure.^56^

$$Wound\;closure\;\%=\left(\frac{the\;area\;of\;wound\;at\;0h-the\;area\;of\;wound\;at\;different\;time\;points}{the\;area\;of\;wound\;at\;0h}\right)\times 100\%$$

#### Immunofluorescence staining

Cultured LSCs grown on cover slides were rinsed with PBS three times, fixed with 4% paraformaldehyde for 20 min, and permeabilized with 0.1% Triton X-100 for 20 min, then blocked with 10% donkey serum in PBS for 20 min at room temperature. After rinsing three times with PBS, the slides were incubated overnight at 4°C with anti-Ki67 (1:200, Abcam), anti-ABCG2(1:100, Santa Cruz), anti-ΔNP63α (1:100, Santa Cruz), anti-CK3 (1:100, Santa Cruz), anti-CK12 (1:100, Santa Cruz). Then the slides were rinsed 3 times with the washing buffer and incubated with Alexa Fluor 488 or 594-conjugated secondary antibodies (1:400, Life Technologies) for 1h at room temperature. 5-ethynyl-2′-deoxyuridine (EdU) staining was performed using a Click-iT cell proliferation kit (Invitrogen) following the manufacturer’s protocol. After extensive washing, DAPI (Invitrogen) was used for nuclear counterstaining. Immunolabelled cells were examined with a fluorescence microscope (Nikon Eclipse 80i; Nikon Instruments, Tokyo, Japan).

For corneal nerve staining, we followed the previously described procedure.^57^ Briefly, whole-mount corneas from each group were fixed in Zamboni fixative solution (Solarbio) for 1 h, followed by permeabilization with 0.1% Triton X-100 for 1h, then blocking with 3% goat serum in PBS for 2 h. Subsequently, the corneas were incubated with tubulin β-III (1:200, Abcam) at 4°C overnight, followed by incubation with Alexa Fluor 594-conjugated secondary antibody (1:400, Life Technologies) at room temperature for 1 h. The corneas were then cut into petal shapes, and the nerve morphology was examined and captured using a fluorescence microscope (Nikon Eclipse 80i; Nikon Instruments, Tokyo, Japan).

#### RT-qPCR

Total RNA was isolated from cultured cells using a Rneasy Mini Kit (Qiagen, Valencia, CA, USA). Quantitative PCR (qPCR) was then performed on RNA samples. A PrimeScript RT reagent kit (TaKaRa, Dalian, China) was used to transcribe RNA into cDNA. Real-time PCR was conducted on an ABI Prism 7000 instrument using SYBR Green PCR Master Mix (Life Technologies). The sequences of the primers used are summarized in Table S1. For normalization of gene expression levels, ratios relative to the housekeeping gene GAPDH were calculated by the ΔΔCT method.

#### Colony-Forming Efficiency assay

Cells were plated onto a six-well culture dish at a density of 1000 per well and cultured for 12 days. The medium and Tregs in the culture were refreshed every 48 h. Three replicates were set for each subgroup. Colonies were fixed with 4% PFA and stained with 0.1% crystal violet. After extensive washing, colonies were counted independently by three investigators. CFE was calculated by the amount of colonies/number of cells seeded × 100.

#### RNA-seq analysis

Total RNA was extracted using the mirVana miRNA Isolation Kit (Ambion) following the manufacturer’s protocol. RNA purity and quantification were evaluated using the NanoDrop 2000 spectrophotometer (Thermo Scientific, USA). RNA integrity was assessed using the Agilent 2100 Bioanalyzer (Agilent Technologies, Santa Clara, CA, USA). The libraries were constructed using TruSeq Stranded mRNA LTSample Prep Kit (Illumina, San Diego, CA, USA) according to the manufacturer’s instructions. Then the libraries were sequenced on the Illumina sequencing platform (Illumina HiSeq X Ten). Raw reads were filtered by Trimmomatic^58^ and then mapped to the human genome (GRCh38) using HISAT2.^59^ FPKM^60^ of each gene was calculated using Cufflinks,^61^ and the read counts of each gene were obtained by HTSeqcount.^62^ Differential expression analysis was performed using the DESeq (2012) R package.^63^ *p* value <0.05 and fold change >2 or <0.5 was set as the threshold for significantly differential expression. Hierarchical cluster analysis of DEGs was performed to demonstrate the expression pattern of genes in different groups and samples. GO enrichment and KEGG^64^ pathway enrichment analysis of DEGs were performed respectively using R based on the hypergeometric distribution. Raw RNA-seq data generated during this study is deposited in GEO and publicly available as of the date of publication. Accession number is: GSE291731.

### Quantification and statistical analysis

The data were presented as mean values ±standard error of the mean (SEM), derived from three separate experiments. To determine differences among multiple groups or between two groups, one-way analysis of variance (ANOVA) or Student’s t test was employed. GraphPad Prism 9 software (La Jolla, CA, USA) was used to facilitate statistical analysis and graphical representation of the data. Significance levels were denoted as: ∗*p* < 0.05, ∗∗*p* < 0.01, ∗∗∗*p* < 0.001, and ∗∗∗∗*p* < 0.0001.
