# Supplementary material for: Therapeutic potential of regulatory T cells for stem cell regulation: Insights from Treg-mediated enhancement of limbal stem cell functions
Source: iScience. 2025 Apr 22;28(5):112515. doi: 10.1016/j.isci.2025.112515 (PMC12146654; doi:10.1016/j.isci.2025.112515)
Supplement: Document S1. Figures S1–S4 and Tables S1 and S2 [file mmc1.pdf]

iScience, Volume 28

Supplemental information

Therapeutic potential of regulatory T cells for stem cell regulation: Insights from Treg-mediated enhancement of limbal stem cell functions

Fei Fang, Tingxi A, Junzhao Chen, Shiding Li, Tianyi Zhou, Liangbo Chen, Yao Fu, and Chunyi Shao

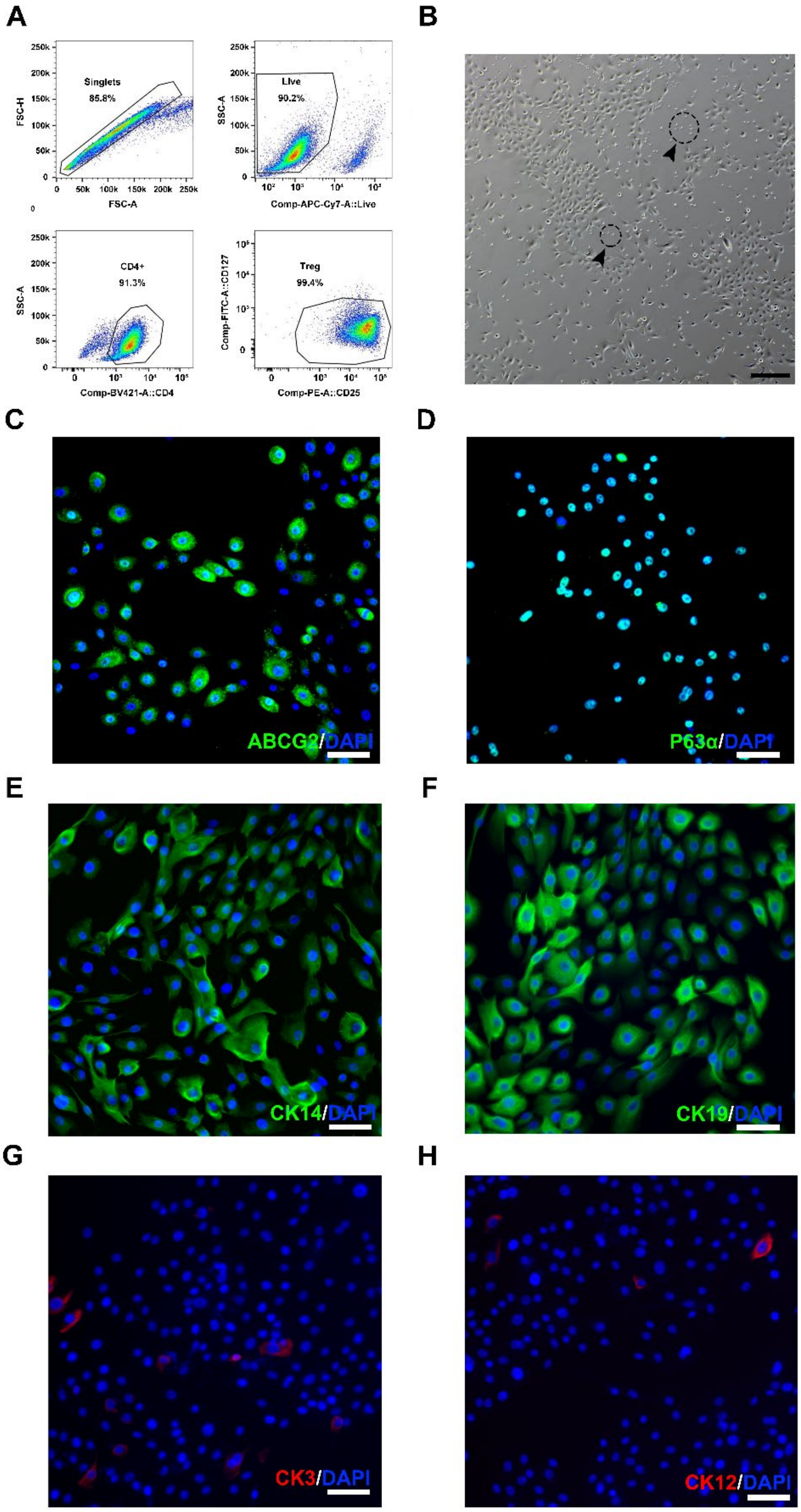

Supplementary Figure 1. Characterization of Regulatory T Cells and Limbal Stem Cells .
(A) Flow cytometry analysis revealed that the purity of CD4^{+}CD25^{+}CD127^{dim} Tregs in viable cells exceeded 90%; **(B)** A microscopic view of co-cultured LSCs and Tregs, where the dashed circles indicate small round cells representing Tregs, while the larger pebble-shaped or short-spindle-shaped cells represent LSCs. Scale bar=200μm; **(C-H)** The immunofluorescence staining of the LSC marker ABCG2 (green), P63α (green), CK14(green), CK19(green), and corneal epithelial cell marker CK3(red) and CK12(red), with the cell nuclei counterstained using DAPI. Scale bar=100μm.

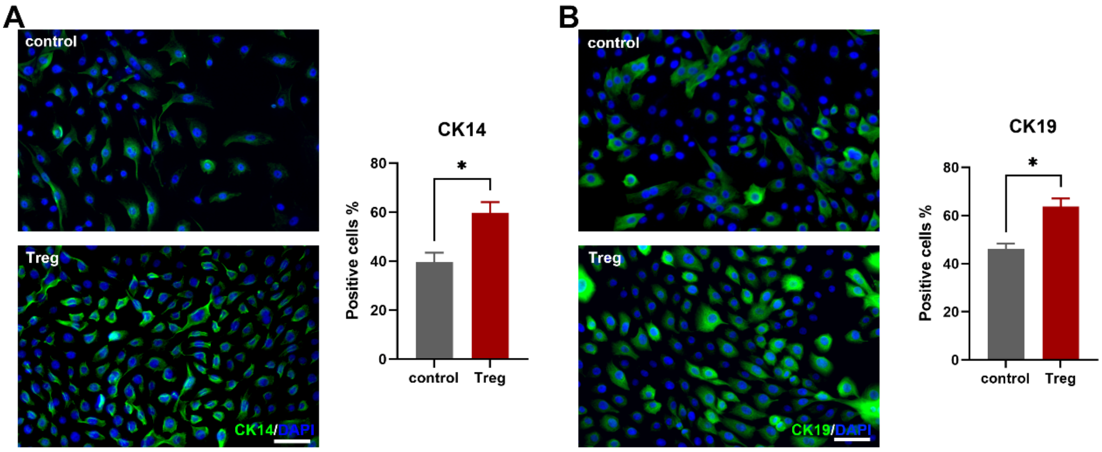

Supplementary Figure 2. Co-culturing with Tregs preserves the stemness characteristics of LSCs. (A,B) Immunofluorescence staining images of CK14 and CK19 of LSCs at 24 hours post co-culture are presented. Nuclei were counterstained with DAPI. Scale bars=100 µm. Data are presented as the mean ± SEM; $*p < 0.05$; $n = 3$

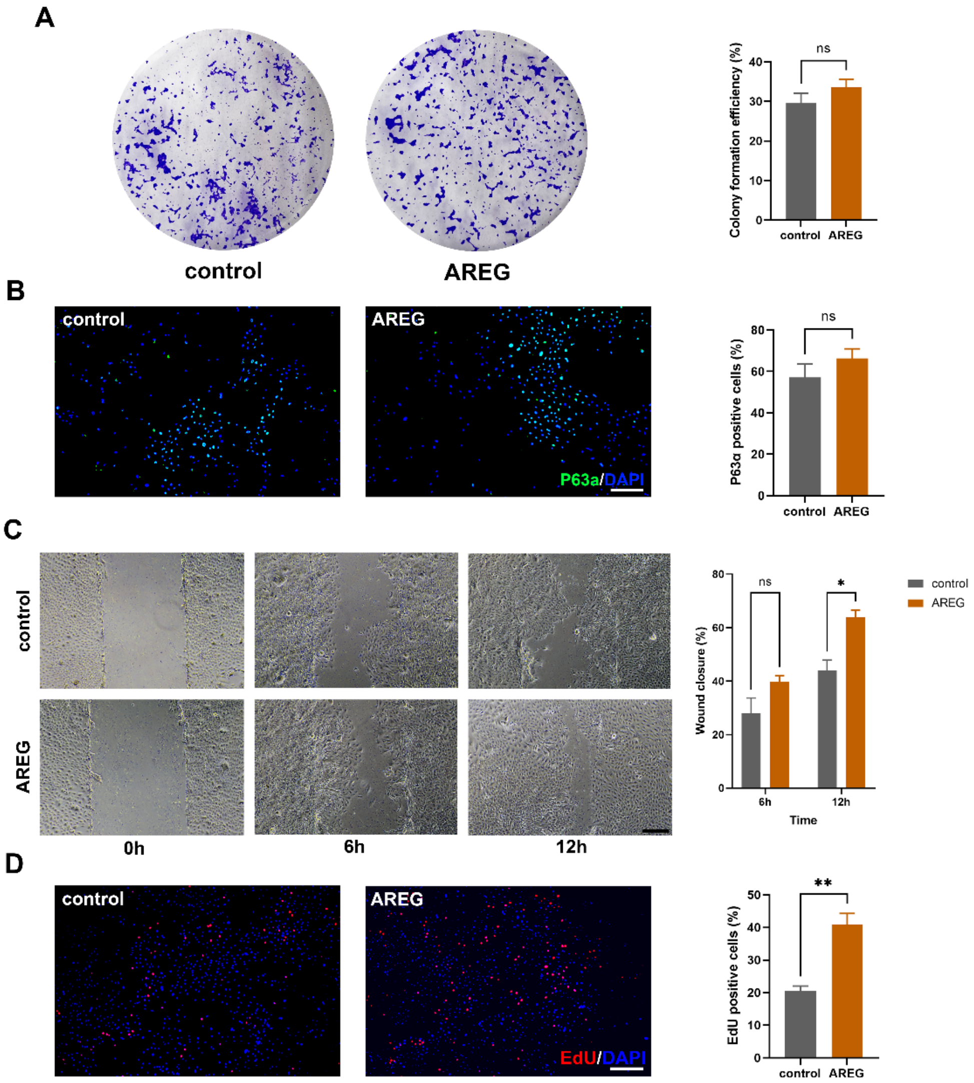

Supplementary figure 3. AREG promotes the proliferation and migration of LSCs, but has no significant effect on maintaining stemness.

(A) Representative images depicting formation of LSC colonies in each group were captured 12 days post-co-culture. **(B)** Immunofluorescence staining images of P63α (green) of different groups of LSCs are presented. The positive cell count chart is located to the right of the fluorescence images. Nuclei were counterstained with DAPI. **(C)** Representative images depicting the scratch assay from both groups were captured at 0, 6, and 12 hours. **(D)** Representative immunofluorescence images of EdU incorporation (red) of LSCs are presented for each group. Nuclei were counterstained with DAPI. Scale bars=100 μm. Data are presented as the mean ± SEM; ns, not significant, *$p < 0.05$, **$p < 0.01$; $n = 3$.

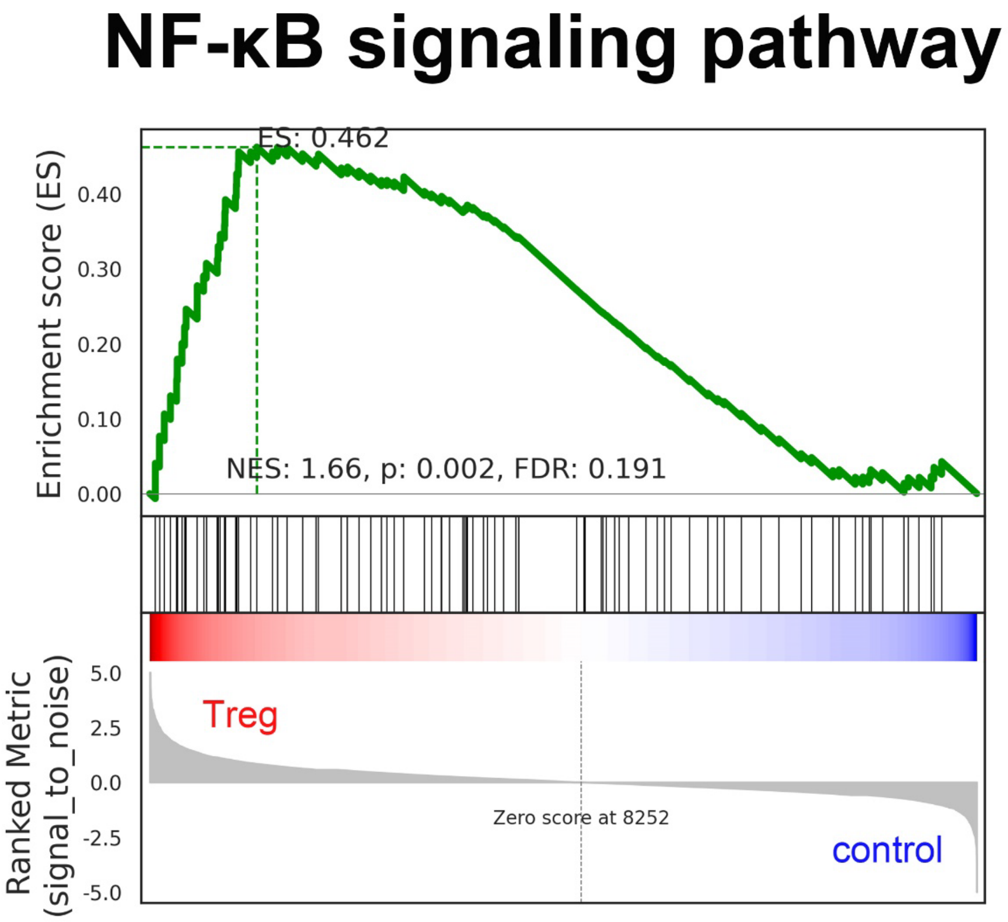

Supplementary figure 4. GSEA analysis results depicted enrichment of differentially expressed genes of LSCs in NF-kB pathway.

Supplementary table 1. Primer sequences for RT-qPCR

Genes (human)	Forward (5’-3’)	Reverse (3’-5’)
**GAPDH**	AGGAGAGTGTTTCCTCGICC	TGCCGTGAGTGGAGICATAC
**ABCG2**	CAGGTGGAGGCAAATCTTCGT	ACCCTGTTAATCCGTTCGTTTT
**TP63**	GGACCAGCAGATTCAGAACGG	AGGACACGTCGAAACTGTGC
**KRT3**	GCAGGGCACAAGTTCCATCT	TCTCTCCCCGAGGATGTTGTC
**KRT12**	CTCTCCTCGCAGAGTGTGATA	AACTAGAACCAAACATGGAAGCA
**MKI67**	ACGCCTGGTTACTATCAAAAGG	CAGACCCATTTACTTGTGTTGGA
**CLDN4**	TGGGGCTACAGGTAATGGG	GGTCTGCGAGGTGACAATGTT
**CXCL16**	CCCGCCATCGGTTCAGTTC	CCCCGAGTAAGCATGTCCAC
**MMP9**	TGTACCGCTATGGTTACACTCG	GGCAGGGACAGTTGCTTCT
**TJP1**	GAGCAGGCTTTGGAGGAGAC	TGGGACAAAAGTCCGGGAAG
**EGFR**	AGGCACGAGTAACAAGCTCAC	ATGAGGACATAACCAGCCACC
**STAT5A**	GCAGAGTCCGTGACAGAGG	CCACAGGTAGGGACAGAGTCT
**VEGFA**	AGGGCAGAATCATCACGAAGT	AGGGTCTCGATTGGATGGCA

Supplementary table 2. Participant characteristics

Healthy donors	Age	Sex	Race/ Ethnicity
**Participant 1**	25	Female	Asian
**Participant 2**	29	Male	Asian
**Participant 3**	25	Female	Asian
**Participant 4**	25	Female	Asian
**Participant 5**	26	Female	Asian
**Participant 6**	26	Female	Asian
**Participant 7**	24	Male	Asian
